# 

**Published:** 1891-12

**Authors:** 


### INDEX TO VOLUME XII.

### ORIGINAL COMMUNICATIONS.

PAGE

The Influence of Adenoid Hypertrophy at the Vault of the Pharynx upon the Development of the Hard Palate. By Dr. D. Bryson Delavan, New York . . . 1
Concerning Third Molars. By Dr. C. A. Brackett, Newport, R. I. . . . 10
Professional Ethics. By Dr. S. B. Bartholomew, Springfield, Massachusetts . . . 17
The Relation of Dental Societies to the Profession. By Jos. King Knight, D.D.S., Boston, Massachusetts . . . 23
A Case of Necrosis of the Superior Maxillary Bone. By B. D. Friedenwald, D.D.S. . . . 29
Inter-Dental Splints. By Alonzo P. Beale, D.D.S., Philadelphia . . . 81
Erosion. By William H. Trueman, D.D.S., Philadelphia . . . 86
Why has the American Dentist a High Reputation throughout the Civilized World? By Dr. C. T. Terry, Milan, Italy . . . 97
Independent Dental Journalism. By L. Ashley Faught, D.D.S., Philadelphia . . . 100
A Case in Practice. By J. G. W. Werner, D.M.D. . . . 105
Some Conditions of Mutual Interest to the Dentist and the Oculist. By George T. Stevens, M.D., New York . . . 145
The Obtunding of Sensitive Dentine. By George F. Eames, M.D., D.D.S. . . . 158
The Function of the Odontoblast. By J. E. Stanton, M.D., D.M.D., Boston, Massachusetts . . . 163
Anæsthetics in Dental Practice. By J. Cowan Woodburn, M.D., Lecturer in Glasgow Dental Hospital . . . 165
Creolin. By Dr. Wm. H. Potter . . . 209
The Retention or Removal of the Sixth-Year Molar,—Which? By Theodore F. Chupein, Philadelphia . . . 213
Some Ancient Theories. By H. H. Schumann, D.D.S., Chicago . . . 218
The Formation of Enamel. By R. R. Andrews, D.D.S. . . . 273
Plastic Materials in the Preparatory Treatment of Teeth. By Jacob L. Williams, M.D. . . . 281
Work. By Professor J. S. Wight, M.D. . . . 284
Office Notes. By Dr. William H. Rollins . . . 292

PAGE
Removal of the Pulp by the Use of Cocaine. By Edward C. Briggs, M.D., D.M.D. . . . . . . . . . . . . . . . . . . . . . . . . . . . . 296
Structure of Protoplasm. By Carl Heitzmann . . . . . . . . . . . . . 341
The Physiology and Pathology of the Second Dentition. By Richard Cole Newton, M.D., Montclair, New Jersey . . . . . . . . . . . . . 353
Aneurismal Tumor of the Right Alveolar Process and Vault of the Mouth treated by Injection. By John S. Marshall, M.D., Chicago, Illinois . . . . . . . . . . . . . . . . . . . . . . . . . . . . . 367
Prophylaxis in the Field of the Dental Surgeon. By Charles B. Atkinson, D.D.S., New York . . . . . . . . . . . . . . . . . . . . . . . 371
Treatment of Proximate Surfaces. By W. H. Dwinelle, M.D., D.D.S. . 376
The Organization and Diseases of the Teeth. By E. G. Tucker, M.D. . . 379
Dental Education. By Henry Leffmann, M.D., D.D.S. . . . . . . . . 383
Immediate Root-Filling. By J. A. Libbey, D.D.S., Pittsburg, Pennsylvania . . . . . . . . . . . . . . . . . . . . . . . . . . . . . 388
Anæsthetic Operation for the Treatment of Diseases of the Antral Chamber. By J. N. Farrar, M.D., D.D.S., New York City . . . . . . . 421
Proper Care of the Eye-Sight. Lewis S. Dixon, M.D. . . . . . . . . . 423
A Cursory Glance at Tetanus. By G. Lenox Curtis, M.D., D.D.S. . . . 438
A Method of Correcting Irregularities. By J. Lehman Eisenbrey, D.D.S. . . . . . . . . . . . . . . . . . . . . . . . . . . . . 444
The Extraction of the First Permanent Molar. By Dr. Eugene J. Wetzel, Mulhouse, Germany . . . . . . . . . . . . . . . . . . . . . . . 446
The Relation between Dental Caries and Diet. By L. Ashley Faught, D.D.S., Philadelphia . . . . . . . . . . . . . . . . . . . . . . . 448
Secret Preparations. By Dr. S. B. Palmer, Syracuse, New York . . . . 451
Local Anæsthesia and the Dentist. By Dwight M. Clapp, D.M.D., Boston, Massachusetts . . . . . . . . . . . . . . . . . . . . . . . . . 452
An Improvement in Flask Presses used within the Vulcanizer. By William H. Trueman, D.D.S., Philadelphia . . . . . . . . . . . . . . . 454
The Injurious Effects of Vegetable and Mineral Acids upon the Enamel of the Human Teeth, with some Curious Illustrations. By George W. Weld, M.D., D.D.S. . . . . . . . . . . . . . . . . . . . . . . 501
Address by Eugene S. Talbot, M.D., D.D.S. . . . . . . . . . . . . . 510
The Teeth of Invertebrate Animals. By Alton H. Thompson, D.D.S., Topeka, Kansas . . . . . . . . . . . . . . . . . . . . . . . . . 519
The Genesis of the Contour Filling. By George S. Allan, D.D.S., New York City . . . . . . . . . . . . . . . . . . . . . . . . . . . 525
A Discussion of the Fee Problem. By William Barker, D.D.S. . . . . 533
Dental Organizations. By Walter Harrison, D.D.S., D.M.D., Brighton, England . . . . . . . . . . . . . . . . . . . . . . . . . . . . 541
The Bacteria of the Air as a Disturbing Factor in Dental and Surgical Operations. By W. D. Miller, M.D., D.D.S., Berlin, Germany . . 581
Growth of the Cementum. By R. R. Andrews, D.D.S., Cambridge Massachusetts . . . . . . . . . . . . . . . . . . . . . . . . . . . 587
Dental Infirmary Patients. The Use and Abuse of Dental Charity. By Richard Grady, M.D., D.D.S., Baltimore, Maryland . . . . . . . . 592
Dental Responsibility for Early Diagnosis of Tumors of the Mouth and Jaws. By Arthur K. Stone, A.M., M.D. . . . . . . . . . . . . . 602

PAGE

A Comparison of the Methods and Surroundings of English and American Dentists. By Waldo E. Boardman, D.M.D., Boston, Massachusetts . . . . . . . . . . . . . . . . . . . . . . . . . . . . 609
Observations on Dental Caries. By Dr. A. M. Ross, Springfield, Massachusetts . . . . . . . . . . . . . . . . . . . . . . . . . . 612
Tincture of Chloride of Iron. By Thomas J. Giblin, D.M.D. . . . . . 614
The Relations and Resources of our Specialty. By Horatio C. Meriam, D.M.D.. Salem, Massachusetts . . . . . . . . . . . . . . . . . . . 617
What Value has Argenti Nitras as a Therapeutic Agent in Dentistry? By Dr. E. A. Stebbins, Shelburne Falls, Massachusetts . . . . . . 661
Pathological Conditions produced by Galvanic Action between Dissimilar Metals in the Treatment of Caries of the Teeth. By George W. Whitefield, M.D., D.D.S., Evanston, Illinois . . . . . . . . . . 671
Vitality as a Germicide. By Thomas Fillebrown, M.D., D.M.D., Boston, Massachusetts . . . . . . . . . . . . . . . . . . . . . . . . . 678
The Rheumatic and Gouty Diathesis as Manifested in Diseases of the Peridental Membrane. By John S. Marshall, M.D. . . . . . . . . . 690
A Few Words in Regard to the Sterilization of Instruments. By Harold C. Ernst, M.D. . . . . . . . . . . . . . . . . . . . . . . . . . 741
Histological Methods and Results. By L. C. Ingersoll, Keokuk, Iowa . 748
Alveolotomy. By G. S. Junkerman, M.D., D.D.S., Cincinnati, Ohio . . 760
A New Method of Running the Suspension Dental Engine by the Electric Current. By T. A. Fletcher, D.D.S., New York . . . . . . . 762
The Chemical Metamorphosis of the Fluids of the Mouth with Relation to Discoloration of Gold. By Edward S. Niles, D.M.D. . . . . . . . . 805
How shall we practise Dentistry in order to make it Healthful? By Dr. Charles T. Terry, Milan, Italy . . . . . . . . . . . . . . . . . 809
Abrasions of the Teeth. By Dr. A. F. Townsend, Worcester, Massachusetts 811
Rapid Operations. By Frank Perrin, D.M.D. . . . . . . . . . . . . 815

### REPORTS OF SOCIETY MEETINGS.

American Dental Association.—Thirtieth Annual Meeting, Excelsior Springs, Missouri, August, 1890 . . . . . . . . . . . . . . 31, 109, 172
New York Odontological Society . . . . . . . 41, 223, 300, 392, 456, 544, 694
American Academy of Dental Science, 55, 182, 236, 319, 399, 466, 558, 631, 728
Harvard Odontological Society . . . . . . . . . . . . 191, 323, 479, 640, 779
Union Dental Meeting, held in Boston, Massachusetts, October, 1890 . . 63
New Jersey State Dental Society.—Twentieth Annual Session, 117, 177, 240, 401, 470
Pennsylvania State Dental Society . . . . . . . . . . . . . 125, 406, 475
Swiss Odontological Society . . . . . . . . . . . . . . . . . . . . 127
The James Truman Dental Society of the University of Pennsylvania . 256
American Medical Association . . . . . . . . . . . . . . . . . 569, 627, 707
Odontological Society of Pennsylvania . . . . . . . . . . . . . . . 307, 786
National Association of Dental Faculties . . . . . . . . . . . . . . . 469
National Association of Dental Examiners . . . . . . . . . . . . . . 652
American Dental Association, Saratoga Springs, New York, August, 1891, 717, 764, 818

### EDITORIALS.

PAGE
American Dental Degrees in England . . . . . . . . . . . . . . . . . . . 66
Koch's Treatment of Tuberculosis . . . . . . . . . . . . . . . . . . . 68
The Tariff on Manufactured Teeth . . . . . . . . . . . . . . . . . . . 130
Dr. (?) Hunter's Dental Anodyne . . . . . . . . . . . . . . . . . . . 131
To Contributors . . . . . . . . . . . . . . . . . . . . . . . . . 132
Have we a Code of Ethics? . . . . . . . . . . . . . . . . . . . . . 198
Independent Dental Journalism and its Use . . . . . . . . . . . . . . . 199
Annual Meeting of the Dental Protective Association . . . . . . . . . . 201
Professional Plagiarism . . . . . . . . . . . . . . . . . . . . . . 259
American Dental Association . . . . . . . . . . . . . . . . . . . 261, 656
Contour Fillings: Their Place in Dental History . . . . . . . . . . . . 332
William H. Atkinson, M.D., D.D.S. . . . . . . . . . . . . . . . . . . 334
The Conventions . . . . . . . . . . . . . . . . . . . . . . . . . 408
The United States Supreme Court Decision in "Tooth-Crowns" . . . . 410
Dr. James W. White . . . . . . . . . . . . . . . . . . . . . . . . 411
Dr. Edward Maynard . . . . . . . . . . . . . . . . . . . . . . . . 411
Arsenious Acid in Dentistry . . . . . . . . . . . . . . . . . . . . . 485
James W. White, A.M., M.D., D.D.S. . . . . . . . . . . . . . . . . . 488
Saratoga Springs, New York . . . . . . . . . . . . . . . . . . . . . 489
Southern Dental Association . . . . . . . . . . . . . . . . . . . . . 489
Editor of the *Dental Cosmos* . . . . . . . . . . . . . . . . . . . . 489
Original Ideas—Inlays . . . . . . . . . . . . . . . . . . . . . . . 575
Joint Union Meeting of the New Jersey and Pennsylvania State Dental Societies . . . . . . . . . . . . . . . . . . . . . . . . . 578
The National Association of Dental Faculties and the National Association of Dental Examiners . . . . . . . . . . . . . . . . . . . . 657
Criticism of Dental Colleges . . . . . . . . . . . . . . . . . . . . 731
Nitrate of Silver.—Stebbins . . . . . . . . . . . . . . . . . . . . 734
To the *Dental Review* . . . . . . . . . . . . . . . . . . . . . . . 734
Deposit Plates . . . . . . . . . . . . . . . . . . . . . . . . . . 735
The Dental Protective Association . . . . . . . . . . . . . . . . . . 798
The *Dental Advertiser* and its New Editor . . . . . . . . . . . . . . 800
Where should Papers be published? . . . . . . . . . . . . . . . . . . 847
The Closing of the Year . . . . . . . . . . . . . . . . . . . . . . 850
Assistant Editor . . . . . . . . . . . . . . . . . . . . . . . . . 851

### BIBLIOGRAPHY.

BIBLIOGRAPHY . . . . . . . . . . . . 69, 132, 201, 261, 335, 489, 735, 800, 852

### OBITUARY.

William W. Fouché, D.D.S. . . . . . . . . . . . . . . . . . . . . . 77
Daniel Frank Whitten, D.M.D. . . . . . . . . . . . . . . . . . . . . 336
William H. Atkinson, M.D., D.D.S. . . . . . . . . . . . . . . . . 336, 417
Dr. Ambler Tees, Sr. . . . . . . . . . . . . . . . . . . . . . . . 336
Dr. Edward Maynard . . . . . . . . . . . . . . . . . . . . . . . . 413

PAGE

Professor Joseph Leidy . . . . . . . . . . . . . . . . . . . . . . 416
James W. White, A.M., M.D., D.D.S. . . . . . . . . . . . . . . . . 490
Chicago Dental Society—Resolutions on the Death of Drs. William H. Atkinson and James W. White . . . . . . . . . . . . . . . . . 492
Resolution of the Central Dental Association of Northern New Jersey on the Death of Dr. Atkinson . . . . . . . . . . . . . . . . . . . 493
St. Louis Dental Society—Resolutions on the Death of Dr. Atkinson . . 494
Dr. Ralph A. Parsons . . . . . . . . . . . . . . . . . . . . . . . 494
Resolutions on the Death of Dr. James W. White . . . . . . . . . . . 578
American Academy of Dental Science—Resolutions of Respect . . . . . 579
C. E. Hawley, D.D.S. . . . . . . . . . . . . . . . . . . . . . . . 579
Dr. Frank Whitten, D.M.D.,—Resolutions of Respect . . . . . . . . . 659
Resolutions of Respect to the Late Dr. William H. Atkinson, of New York . . . . . . . . . . . . . . . . . . . . . . . . . . . . . 660
C. A. Kingsbury, M.D., D.D.S. . . . . . . . . . . . . . . . . . . . 801
Dr. David Roberts . . . . . . . . . . . . . . . . . . . . . . . . . 803
American Dental Association—In Memoriam: Dr. W. H. Atkinson . . 855

### DOMESTIC CORRESPONDENCE.

DOMESTIC CORRESPONDENCE . . . . . . . . . . . 138, 205, 412, 660, 737, 857

### FOREIGN CORRESPONDENCE.

FOREIGN CORRESPONDENCE . . . . . . . . . . . . . . . . . . . . . 204

### CURRENT NEWS.

CURRENT NEWS . . . . . . 78, 142, 205, 263, 338, 419, 495, 580, 739, 804, 861

SUBSCRIBE FOR THE

### International Dental Journal

**The Organ of the Profession,**

PUBLISHED BY THE

### INTERNATIONAL DENTAL PUBLICATION COMPANY:

**LOUIS JACK,** *President,* Philadelphia.
**BENJAMIN LORD,** *Vice President,* New York.
**C. N. PEIRCE,** *Treasurer,* Philadelphia.
**GEO. S. ALLAN,** *Secretary,* 51 W. 37th St., New York.

---

*PUBLICATION OFFICE:*

**716 Filbert Street, Philadelphia, Pa.**

---

**FOREIGN CORRESPONDENTS:**

Dr. ARKÖVY, Budapesth, Hungary.
W. GEO. BEERS, D.D.S., Ed. Dominion Dental Journal, Montreal.
L. C. BRYAN, D.D.S., Basel.
ALFRED BURNE, D.D.S., Sydney, New South Wales.
F. BUSCH, M.D., Berlin.
GEORGE CUNNINGHAM, Cambridge, Eng.
I. B. DAVENPORT, D.D.S., Paris.
PAUL DUBOIS, Ed. L'Odontologie, Paris.
WILLIAM DUNN, Jr., L.D.S., Florence.
A. H. EDWARDS, D.D.S., Madrid.
W. ST. GEORGE ELLIOTT, M.D., D.D.S., Lon.
OSWALD FERGUS, D.D.S., Glasgow, Scotland.
ALPHONSE FEUCHEL, Zahnarzt, St. Petersburg.
C. R. FLETCHER, D.D.S., Sao Paulo, Brazil.
WALTER HARRISON, L.D.S., D.M.D., Brighton, Eng.
ROBERT S. IVY, D.D.S., Shanghai, China.
N. S. JENKINS, D.D.S., Dresden.
F. KLAPPROTH, Zahnarzt, St. Petersburg.
L. KOLBE, " " "
Dr. GEO. P. E. KUHN, Paris.
W. BOWMAN MACLEOD, L.D.S., Edinburgh.
W. D. MILLER, Ph. D., M.D., D.D.S., Berlin.
WILLIAM SACHS, D.D.S., Breslau.
J. G. VAN MARTER, B.A., D.D.S., Rome.
J. M. WHITNEY, M.D., D.D.S., Honnolula.
LUDWIG WARNEKROS, Zahnarzt, Berlin.
LUDW. ADOLPH WEIL, M.D., Munich.
J. COWAN WOODBURN, M.D., Glasgow.

---

STOCKHOLDERS:

Frank Abbott,
Geo. S. Allan,
W. W. Allport,
R. R. Andrews,
H. A. Baker,
A. E. Baldwin,
W. C. Barrett,
A. P. Beale,
S. T. Beale, Jr.,
C. S. Beck,
G. V. Black,
C. F. W. Bödecker,
E. A. Bogue,
W. G. A. Bonwill,
C. A. Brackett,
Thomas Bradley,
Edward C. Briggs,
Albert H. Brockway
A. E. Brown,
Wm. Carr,
Geo. H. Chance,
G. F. Cheney,
Dwight M. Clapp,
John D. Codman,
Chas. D. Cook,
J. N. Crouse,
Edw. T. Darby,
H. S. Draper,
Wm. H. Dwinelle,
A. R. Eaton,
Chas. J. Essig,
J. N. Farrar,
T. Fillebrown,
W. B. Finney,
T. A. Fletcher,
M. W. Foster,
Chas. E. Francis,
E. C. Fundenberg,
W. F. Fundenberg,
W. H. Fundenberg,
E. S. Gaylord,
J. P. Geran,
A. H. Gilson,
S. H. Guilford,
John I. Hart,
O. E. Hill,
J. F. P. Hodson,
J. Morgan Howe,
Robert Huey,
A. O. Hunt,
Louis Jack,
V. H. Jackson
C. R. Jefferis,
C. A. Kingsbury,
Edw. C. Kirk,
C. R. E. Koch,
W. T. La Roche,
W. K. Leech,
F. A. Levy,
Wilbur F. Litch,
J. Bond Littig,
Benjamin Lord,
Geo. W. Lovejoy (Canada),
John S. Marshall,
H. J. McKellops,
Charles McManus,
James McManus,
Dan'l Neal McQuillan,
Horatio C. Meriam,
W. D. Miller (Berlin),
W. E. Page,
S. B. Palmer,
C. B. Parker,
D. D. Peabody,
C. N. Peirce,
S. G. Perry,
W. A. Phreaner,
Chas. E. Pike,
W. E. Pinkham,
W. H. Potter,
Chas. P. Pruyn,
H. C. Register,
F. A. Roy,
Z. T. Sailer,
L. D. Shepard,
Geo. R. Shidle,
Eugene H. Smith,
B. Holly Smith,
H. A. Smith,
S. G. Stevens,
W. X. Sudduth,
Eugene S. Talbott,
Ambler Tees,
J. D. Thomas,
James Truman,
Wm. H. Trueman,
W. W. Walker,
I. F. Wardwell,
G. W. Warren,
T. S. Waters,
Geo. W. Weld,
Julius G. W. Werner,
Jacob L. Williams,
R. B. Winder,
C. A. Woodward,
J. A. Woodward,
W. A. Woodward,
W. J. Younger.

---

***Subscription Price, $2.50 per Year.***

